# Diagnosing confounded Bateman gradients

**DOI:** 10.1093/evolut/qpaf127

**Published:** 2025-06-09

**Authors:** Krish Sanghvi, Jonathan M Henshaw, Alex Kacelnik, Tim Janicke, Irem Sepil

**Affiliations:** Department of Biology, University of Oxford, Oxford, United Kingdom; Institute of Biology, University of Freiburg, Freiburg, Germany; Department of Biology, University of Oxford, Oxford, United Kingdom; CNRS Centre for Functional and Evolutionary Ecology, Montpellier, France; Department of Biology, University of Oxford, Oxford, United Kingdom

**Keywords:** anisogamy, cryptic female choice, polyandry, sexual selection, sperm competition, theoretical biology

## Abstract

The Bateman gradient is a fundamental metric of sexual selection, often interpreted as the fitness advantage individuals gain by increasing their number of mates. However, it is recognized that any traits influencing both mating and reproductive success can confound the gradient, misrepresenting the strength of precopulatory sexual selection. Yet, the magnitude of this misrepresentation across different biological systems (e.g., differing in anisogamy or strength of sperm competition), which covariates are most problematic, or how confounded relationships can be diagnosed to better interpret the Bateman gradient, remains largely unexplored. To address these gaps, we simulate 9 plausible biological scenarios where the effect of male mating success on reproductive success is confounded. We find that covariances between male mating success and female fecundity or egg allocation confound male Bateman gradients more strongly than covariances between male mating success and ejaculate traits. These differences in the impact of male–female vs. male–male covariances are especially pronounced in systems with high levels of anisogamy and no sperm competition. We provide guidelines for empiricists to visually identify such covariances by recording mating order, and suggest that researchers explicitly state causal assumptions when interpreting Bateman gradients. Additionally, when the covariate is a confounder, not a mediator, we demonstrate that partial Bateman gradients allow better interpretation of the strength of precopulatory sexual selection. These insights into the mechanisms driving variation in the Bateman gradient allow us to clarify its meaning, identify scenarios where its interpretation might be problematic, and offer practical solutions to address this.

## Introduction

The Bateman gradient (Bateman, 1948; henceforth BG or ${{\beta }_{\mathrm{ss}}}$) is a cornerstone of sexual selection theory (Arnold & Duvall, 1994; Henshaw & Jones, 2019). Typically, the BG is calculated as the slope of an ordinary least square (OLS) linear regression of the relativized number of offspring produced [i.e., reproductive success (RS)] on the relativized number of mates [i.e., mating success: (MS)] (Anthes et al., 2017; Arnold, 1994; Jones, 2009; Wade & Shuster, 2005). The BG is used to interpret the selective fitness advantage gained from intrasexual competition for mates. Due to males being less gamete-limited than females, male BGs are typically positive, and steeper than those of females (Janicke et al., 2016), with greater variance in male RS and MS compared with females. These observations have led to predictions of stronger strength of and opportunity for precopulatory sexual selection on males than females. Multiple studies on wild (e.g., Mills et al., 2007; Mobley & Jones, 2007; Rios-Cardenas, 2005; Rodriguez-Munoz et al., 2010; Schulte-Hostedde et al., 2004; Webster et al., 2007) and lab animals (e.g., Andrade, 2005; Davies et al., 2023; Fritzsche et al., 2013; Jones et al., 2002, 2004; Lorch et al., 2008; McDonald et al., 2025; Pelissie et al., 2012), humans (Borgerhoff & Ross 2019), as well as plants (Tonnabel et al., 2019) have employed the BG to interpret precopulatory sexual selection. However, not all studies agree with these predictions (e.g., Apakupakul et al., 2015; Douglas et al., 2020; Gopurenko et al., 2007; Jones et al., 2000; Nason & Kelly, 2020). Furthermore, among other issues, the BG is known to misrepresent the strength of sexual selection when the relationship between MS and RS is confounded by other variables (Anthes et al., 2017; Arnold, 1994; Collet et al., 2014; Fromonteil et al., 2023; Klug et al., 2010; Tang-Martinez, 2005; 2016).

**Figure 1.**
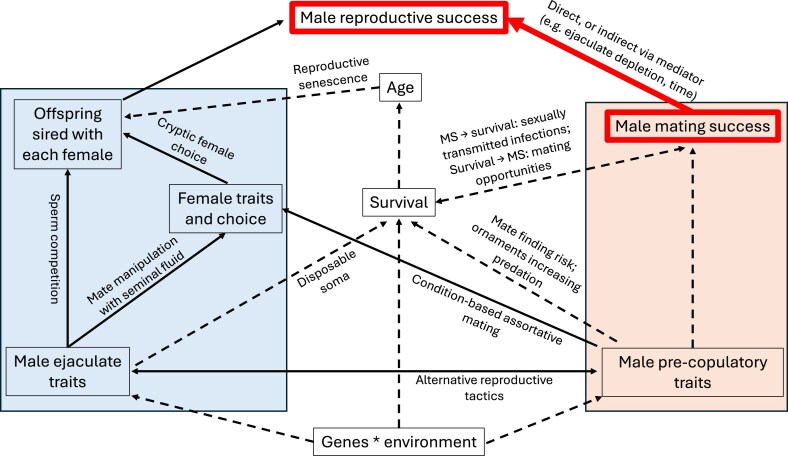
Plausible hypothetical relationships of how male mating success (MS), other traits, and reproductive success (RS) might interact. The Bateman gradient (BG; in red) measures the relationship between MS and RS, typically interpreted as precopulatory selection via traits enhancing MS. However, this interpretation of the BG can be misleading under covariances between MS and other male or female traits that confound the effect of MS on RS. Distinguishing confounding from causal effects (i.e., indirect causal effects via mediators or direct causal effects of MS on RS) is crucial for interpretation of sexual selection. Boxes inside the orange highlighted areas are typically interpreted as precopulatory sexual selection; inside the blue areas interpreted as postcopulatory sexual selection. Arrows show likely direction of causality, and can represent positive or negative effects. Solid arrows are simulated in our study, dashed arrows are not. Examples of each relationship are provided besides arrow.

Within a sex, the BG measures the statistical relationship between an individual’s MS and RS, often interpreted as the potential fitness benefit gained by acquiring more mates. However, there are few empirical tests that experimentally manipulate MS to test its causal influence on RS (but see Andrade & Kasumovic, 2005). Instead, most studies, including Bateman’s (1948) original study, use natural variation in MS and RS to assess their relationship (e.g., Mobley & Jones, 2007; Rios-Cardenas, 2005; Rodriguez-Munoz et al., 2010; Schulte-Hostedde et al., 2004; Webster et al., 2007). Here, however, the correlation between MS and RS is known to not just reflect the causal effect of MS on RS, but also to include the effects of other variables. In males, such confounding could arise due to covariances between male MS and female quality or postcopulatory choice, or between male precopulatory and postcopulatory traits (Anthes et al., 2010; Collet et al., 2012; Davies et al., 2023; Devigili et al., 2015; Evans & Garcia-Gonzalez, 2016; Henshaw et al., 2018; Krakauer, 2008; McDonald & Pizzari, 2016; McDonald et al., 2025; Marie-Orleach et al., 2024; Parker & Tang-Martinez, 2005; Pelissie et al., 2014; Figure 1).

Across animals, various types of confounding can potentially distort the interpretation of sexual selection when using the BG. For example, dominant males (who generally have higher MS than subordinates) may mate with more fecund (Collet et al., 2014; Gerlach et al., 2012; Kraak & Bakker, 1998) or less promiscuous females (Greenway et al., 2021), allowing them to achieve higher RS partly due to female quality or promiscuity, rather than solely because they mate with more females. Likewise, females preferring sperm of dominant compared with subordinate males (Dean et al., 2011; Pizzari & Birkhead, 2000) could lead to dominant males gaining higher RS partly due to the confounded influence of their higher paternity share. Similarly, females allocating a larger proportion of their available eggs to be fertilized by dominant males could produce a confounded positive correlation between MS and RS (Horvathova et al., 2012; Skinner & Watt, 2007). Male MS could also covary negatively with male ejaculate size (Gowaty & Hubbell, 2005), such as in species with alternative male reproductive tactics where one morph mates with more females but allocates smaller ejaculates to each female compared with the other (e.g., Alonzo & Warner, 2000; Durant et al., 2016; Froman et al., 2002; Gage et al., 1995; Klaus et al., 2011; Kvanermo & Simmons, 2013; Lupold et al., 2014; Parker et al., 2013; Preston et al., 2001; Saunders & Shuster, 2020; Warner et al., 1995). Such negative covariances would lead to underestimating the fitness benefits gained by increasing the number of mates, due to different males of different morphs/status having similar RS despite their different MS (reviewed in Da Silva et al., 2024). Male precopulatory and postcopulatory traits could also covary positively due to condition dependence (e.g., Bath et al., 2023; De Nardo et al., 2021; Devigili et al., 2013; Janicke & Chapuis, 2016; reviewed in Simmons et al., 2017), where individuals in higher condition not only gain more mates, but also produce larger or higher quality ejaculates than individuals in lower condition (Hare & Simmons, 2022; Janicke et al., 2015; Mehlis et al., 2015; Morimoto et al., 2016), leading to a confounded relationship between MS and RS. Quantifying the relative influence of specific confounding variables (e.g., female quality vs. male postcopulatory traits) is crucial for interpreting the strength of precopulatory sexual selection. Some statistical methods, such as partial regressions, can reduce bias due to confounding in estimates of selection gradients (discussed in Anthes et al., 2017; Henshaw et al., 2018, 2022). However, other qualitative approaches, such as recording the specific mating order in which individuals encounter mates might be useful too (e.g., Sanghvi et al., 2025b), yet their use has been limited due to the lack of theory on how to interpret BGs based on patterns of offspring production through mating sequences.

Understanding the total causal effect of MS on RS (including both direct and mediated pathways) is key to quantifying the strength of sexual selection. Our simulations explore how confounding obscures this relationship and how statistical approaches might help isolate the causal component (Figure 1). We constructed biologically plausible scenarios to address the following aims: (1) quantify the *magnitude* by which male BG are modulated by different biologically relevant confounders, and test whether different types of confounders (e.g., ejaculate trait vs. female quality) modulate the BG in distinct ways; (2) assess whether some biological systems might be more susceptible to confounded BG than others; (3) demonstrate the importance of recording the mating order of individuals to assess the magnitude and type of confounding; (4) provide guidelines for how partial regressions (following Henshaw et al., 2018) could be used in the presence of confounding variables (without adjusting for mediating variables), to interpret the strength of sexual selection. To address aim (2), we model varying degrees of anisogamy to test whether the potential for confounding on BGs differs between high and low anisogamy systems (Henshaw et al., 2022; Janicke, 2024; Jones et al., 2002; Lehtonen, 2022; Lehtonen & Parker, 2024), and whether sperm competition can modulate this. While our models focus on males, the principles highlighted herein are applicable to females too.

## Methods

### Simulation overview

We used agent-based simulations to construct nine scenarios involving biologically plausible covariances between male MS and various other male or female reproductive traits that can confound the effect of MS on RS. We then quantified the impact of each imposed covariance on the BG. We modeled these across four different biological systems corresponding to each combination of high or low anisogamy, with or without sperm competition. We simulated the number of offspring produced by individual focal males interacting sequentially with multiple females, and subsequently calculated male BGs. All analyses were conducted in R.v.4.3.2 (R core team, 2020). Detailed descriptions of all scenarios are provided below (see the “Simulated scenarios” section). In all simulations, male RS was modeled to depend on the following traits (also see Table 1):

**Table 1.** Mean values for creating normal distributions used for sampling each trait in our simulations for focal males and females, and the rationale for using these. To allow comparison between the low and high anisogamy systems, we retained the same trait values and distributions for all variables in both anisogamy systems, except for starting ejaculate size (*Q*) and number of eggs (*F*).

Trait	Mean value	Rationale
Male mating success (MS)	8	Male fruit flies can mate with up to 10 females over a period of 24 hr, before they run out of their ejaculate reserves (Sanghvi et al., 2025a, 2025b). Under scenarios where mates are not limited, male *Drosophila melanogaster* can mate with up to 11 females, with an average number of mates being 5 to 8 females (Billeter et al., 2012; Douglas et al., 2020). Male junglefowl can mate between 6 and 40 times in quick succession with as many as 5 different females (Alvarez-Fernandez et al., 2019; Pizzari et al., 2003).
Starting ejaculate size (*Q*)	6,000 for low anisogamy 10,000,000 for high anisogamy	Young virgin *D. melanogaster* males have 5,000–8,000 sperm on average (Sanghvi et al., 2025b). Male junglefowl have between a few million to tens of millions of sperm in an ejaculate (Rakha et al., 2015).
Proportion of stored sperm allocated (*T*)	30%	Young virgin *D. melanogaster* males transfer 1,000–2,500 sperm on average to a female (Garbaczewska et al., 2013; Hopkins et al., 2019; Lüpold et al., 2011; McCartney et al., 2021; Sanghvi et al., 2025b). Junglefowl males transfer between 10% and 25% of their ejaculate to each female (Alvarez-Fernandez et al., 2019; Pizzari et al., 2003).
Ejaculate potency (*V*)	70%	Sperm viability and motility varies between 10% and 90% in male *D. melanogaster* (Decanini et al., 2013; Guo et al., 2021; Holman, 2009; Radhakrishnan & Fedorka, 2011; Snook & Hosken, 2004; Lüpold et al., 2011) and between 70% and 90% in the red junglefowl (Rakha et al., 2015).
Sperm retained by females (*L*)	50%	Female *D. melanogaster* retain 400 to 800 sperm in their sperm storage organs for fertilization, after a mating (Avila et al., 2015; Bloch Qazi et al., 2010; McDonough-Goldstein et al., 2022; Sanghvi et al., 2025b; Sepil et al., 2020).
Number of eggs (*F*)	800 for low anisogamy 100 for high anisogamy	A single female *D. melanogaster* can lay up to 800 eggs over a few weeks period, and can use stored sperm from a single mating to fertilize these eggs. When mated to a single male, females can produce up to 400 offspring (Letsinger et al., 1985; Linklater et al., 2007; Zimmering, 1968) over a span of 2–3 weeks. A female domestic fowl can fertilize 20–30 eggs over a period 2–4 weeks, with sperm from a single mating (Chai et al., 2024; Nalbandov & Crad, 1943; Warren & Kilkpatrick, 1929).
Proportion of eggs allocated (*P*)	12.5% per mated male (i.e., 25% for system with sperm competition)	To satisfy Fisher’s condition.

#### Male traits

- Male MS: the total number of females a male mated with. MS is often determined by the expression of male weaponry, male lifespan, male ornamentation, mate coercion ability, energetic and physiological mating costs, courtship ability, or female choosiness. However, we did not simulate these underlying traits explicitly.
- Starting ejaculate size (*Q*): the number of sperm a male has available in his ejaculate reserves before encountering any females. The starting ejaculate size could also represent starting seminal fluid quantity.
- Ejaculate potency (*V*): the proportion of sperm in the male’s ejaculate that has the capacity to fertilize an egg and produce offspring due to intrinsic sperm quality of the donor. Biologically, *V* might represent sperm viability, egg penetration ability, or sperm DNA integrity. $1 - V$ is the proportion of a male’s sperm incapable of fertilization or offspring production.
- Ejaculate allocation (*T*): the proportion of a male’s remaining sperm that he transfers to a given female in his mating sequence when copulating.

#### Female traits

- Number of eggs (*F*): the number of eggs a female has available that are viable and can produce offspring, if fertilized.
- Proportion of eggs allocated (*P*): the proportion of her available eggs the female allocates to be fertilized by the current male she is copulating with.
- Sperm retained (*L*): the proportion of a male’s inseminated sperm that a female uses (i.e., does not eject or absorb, and retains for fertilization). In some cases, this trait could also be a function of a sperm’s ability to enter female sperm storage organs.

### Simulated scenarios

In the null scenario, the relationship between MS and RS was causal, and male MS did not covary with any other male or female traits. In each of the remaining eight scenarios, male MS covaried positively or negatively with one of the male or female traits described above. The difference in the BG between the null scenario and any covariance scenarios reflects the correlation between MS and RS arising from imposed confounding effects. This difference represents the extent to which the strength of precopulatory sexual selection for mates might be misinterpreted. When simulating covariances between male MS and another trait, we assumed that only the distributions of MS and the trait of interest covaried. All other (noncovarying) traits were sampled using a Monte–Carlo process from independent, normal distributions of realistic biological values from two species (*Drosophila melanogaster* and *Gallus gallus*) representing two extreme degrees of anisogamy (see Table 1). This was done as follows:


(1) \begin{eqnarray*}
{\mathrm{trai}}{{{\mathrm{t}}}_i}{\mathrm{\ }}\sim\mathcal{N}({{{\mathrm{\mu }}}_{{\mathrm{trait}}}},\sigma _{{\mathrm{trait}}}^2).
\end{eqnarray*}

When MS covaried with another trait, these two traits were drawn from a bivariate normal distribution of the form


(2) \begin{eqnarray*}
\left[ {\begin{array}{c}{{\rm MS}_{i}}\\ {{\rm trait}_{i}} \end{array}} \right] \sim \mathcal{N} (\mu ,{\mathrm{\Sigma }})
\end{eqnarray*}

with a mean of $$\mu = [{\begin{array}{@{}*{1}{c}@{}} {{{\mu }_{{\mathrm{MS}}}}}\\ {{{\mu }_{{\mathrm{trait}}}}} \end{array}}]$$ and a variance–covariance matrix given by


(3) \begin{eqnarray*}
{\mathrm{\Sigma }} = \left[ {\begin{array}{@{}*{2}{c}@{}} {\sigma _{{\mathrm{MS}}}^2}&{r{{\sigma }_{{\mathrm{MS}}}}{{\sigma }_{{\mathrm{trait}}}}}\\ {r{{\sigma }_{{\mathrm{MS}}}}{{\sigma }_{{\mathrm{trait}}}}}&{\sigma _{{\mathrm{trait}}}^2} \end{array}} \right].
\end{eqnarray*}

For positive and negative covariances, the correlation coefficient (*r*) was set to +0.707 or −0.707, respectively (to give an R^2^ of 0.5). In all our simulations, we recorded the order in which females encountered the focal male, and the numbers of offspring the male produced with each female in his mating sequence. We simulated the following scenarios:

#### Null scenario

In the null scenario (H0), all male and female traits (MS, *Q, V, T, F, P*, and *L*) were sampled independently without any covariances between these traits.

#### Positive covariances due to condition dependence

We simulated two scenarios in which males who are better at acquiring mates also have larger ejaculate reserves [scenario H1.1: ${\mathrm{Cov}}( {{\mathrm{MS}},Q} )\ > 0$] or a higher proportion of potent sperm [scenario H1.2: ${\mathrm{Cov}}( {{\mathrm{MS}},V} )\ > 0$]. Such positive covariances could occur due to condition dependence, where males in better condition have improved precopulatory and postcopulatory sexual traits compared with males in poorer condition.

#### Positive covariance due to male–female interactions

We simulated three scenarios in which MS covaried with female traits. First [scenario H2.1: ${\mathrm{Cov}}( {{\mathrm{MS}},F} )\ > 0$], we created positive covariance between MS and female egg number (*F*). Such a scenario could arise when males who are better at acquiring females also mate with more fecund females. Next [scenario H2.2: ${\mathrm{Cov}}( {{\mathrm{MS}},P} )\ > 0$], we simulated a scenario where MS positively covaried with female allocation of eggs (*P*), for instance, if males who are better at acquiring females are also better at stimulating female into allocating a higher proportion of eggs for fertilization. Third [scenario H2.3: ${\mathrm{Cov}}( {{\mathrm{MS}},L} )\ < 0$], we simulated positive covariance between MS and the proportion of the male’s inseminated sperm that the female retains ($L$). This scenario could arise if females bias sperm retention toward dominant males (who are better at acquiring mates), or if dominant males produce ejaculates that more effectively lead to their sperm being used by the female or entering female sperm storage organs.

Future studies could additionally simulate scenarios where MS negatively covaries with female polyandry, e.g., when males better at acquiring females are also better at suppressing polyandry (or alternatively, males better at acquiring females are more likely to mate with virgin females).

#### Negative covariances due to trade-offs

We simulated negative covariances of male MS with male ejaculate size (*Q*), ejaculate potency (*V*), and the proportion of his stored ejaculate (*T*) that he transfers to a female [scenarios H3.1: ${\mathrm{Cov}}( {{\mathrm{MS}},Q} )\ < 0$; H3.2: ${\mathrm{Cov}}( {{\mathrm{MS}},V} )\ < 0$; scenario H4.1: ${\mathrm{Cov}}( {{\mathrm{MS}},T} )\ < 0$, respectively]. Such scenarios could arise when males, due to resource limitation, face a trade-off between allocating energy toward maintenance/development of precopulatory traits (such as ornamentation or weapons) that improve MS, or toward postcopulatory traits such as producing sperm or maintaining sperm quality. These trade-offs often manifest in species with alternative reproductive tactics, in which dominant males that monopolize females have less viable or fewer sperm, or ejaculate smaller proportion of their stored sperm, compared with subordinate males.

### Simulation structure

#### Without sperm competition

The RS (${\mathrm{R}}{{{\mathrm{S}}}_i}$) of the *i*th male was the sum of all offspring produced by this male across his mating sequence, and was given by


(4) \begin{eqnarray*}
{\mathrm{R}}{{{\mathrm{S}}}_i} = \mathop \sum \limits_{k = 1}^{\mathrm{ M}{{\mathrm{ S}}_i}} {{O}_{ik}},
\end{eqnarray*}

where ${\mathrm{M}}{{{\mathrm{S}}}_i}$ is the male’s MS, and *O_ik_* is the number of offspring he produced with the *k*th female he mated with. When there was no sperm competition, the number of offspring produced by the *i*th male with his *k*th mate was calculated as the minimum of (1) the number of sperm capable of effective fertilization that the *i*th male transfers to the *k*th female that the female retained (${{S}_{ik}}$), and (2) the number of eggs capable of being fertilized that the *k*th female allocated to being fertilized by the *i*th male (${{E}_{ik}}$):


(5) \begin{eqnarray*}
{{O}_{ik}} = {\mathrm{min}}\ ({{S}_{ik}},{{E}_{ik}}).
\end{eqnarray*}

${{S}_{ik}}$ was further partitioned as


(6) \begin{eqnarray*}
{{S}_{ik}}{\mathrm{\ }} = {{N}_{ik}} \cdot {{V}_i} \cdot {{T}_i} \cdot {{L}_{ik}},
\end{eqnarray*}

where ${{N}_{ik}}$ was the number of remaining sperm the male had stored in his ejaculate reserves when encountering female *k*th, ${{V}_i}$ was the proportion of the male’s sperm that were potent/capable of efficient fertilization, ${{T}_i}$ was the proportion of his remaining sperm that the male allocated to the *k*th female, and ${{L}_{ik}}$ was the proportion of inseminated sperm that the female retained for fertilization. The distribution of ${{S}_{ik}}$ values was later used in the models with sperm competition (more below). The number of sperm remaining in the *i*th male’s reserves when encountering the *k*th female (${{N}_{ik}}$) was calculated as his starting ejaculate size before he began mating sequentially with any females (${{Q}_i}$), minus the number of sperm he had already transferred to the females he copulated with before encountering female *k*.


(7) \begin{eqnarray*}
{{N}_{ik}} = {{Q}_i}{{\left( {1 - {{T}_i}} \right)}^{k - 1}}.
\end{eqnarray*}

In females, the number of eggs that the *k*th female allocated for being fertilized by the *i*th male, was calculated as the number of eggs this female had when encountering the male (${{F}_{ik}})\ $ multiplied by the proportion of those eggs which she allocated for being fertilized by the encountered male (${{P}_{ik}})\!:$


(8) \begin{eqnarray*}
{{E}_{ik}}{\mathrm{\ }} = {{F}_{ik}} \cdot {{P}_{ik}}.
\end{eqnarray*}

Fisher’s condition states that the total RS of males and females must be equal (Jennions & Fromage, 2017). At equal sex ratios, this means that the mean RS of males and females must be equal. Because our simulation focused on focal males achieving an average of ${{\mu }_{{\mathrm{MS}}}}$ matings, but females only mated once (or twice with sperm competition) without their mating history being explicitly modeled, Fisher’s condition was not an emergent property of our model. Thus, to ensure this condition was met, we parametrized the mean of *P* (${{\mu }_P}$) as


(9) \begin{eqnarray*}
{{\mu }_P} = \ \frac{1}{{\ {{\mu }_{{\mathrm{MS}}}}}}.
\end{eqnarray*}

Here, the mean proportion of her eggs that a female allocated to be fertilized by a male (${{\mu }_P})$ equalled the inverse of mean MS of males (${{\mu }_{{\mathrm{MS}}}})\ $ in the population. Our models without sperm competition did not necessarily imply the lack of polyandry, and could also reflect systems where females use sperm from multiple mates sequentially.

#### With sperm competition

Next, we simulated the nine scenarios (H0 to H4.1) in a system where each *k*th female in the *i*th male’s mating sequence remated once with another male, right after mating with the *i*th male but before fertilizing any eggs. These models represented a fixed intensity of sperm competition and did not incorporate male strategic ejaculation in response to sperm competition risk. The outcome of sperm competition was determined by a “fair raffle” (Parker et al., 2013), based on the relative numbers of potent sperm of each male retained by each female. The number of potent sperm retained by the female from this second, competing male was sampled from the distribution of ${{S}_{ik}}$​ values of the focal males from the null scenario without sperm competition (i.e., see Equation 6). This distribution of ${{S}_{ik}}$​ values (${{\mathcal{D}}_S}$) represents the number of potent sperm a female retains after mating with a randomly chosen male at a random point in his mating sequence, in the absence of covariances between male MS and any other male or female traits (see Equation 1). We generated ${{\mathcal{D}}_S}$ separately for systems with low and high anisogamy (Supplementary Figure S1). The number of potent sperm from the second male that the female retains (${{A}_{ik}}$) was therefore sampled as


(10) \begin{eqnarray*}
{{A}_{ik}} \sim {{\mathcal{D}}_S}.
\end{eqnarray*}

The paternity share of the *i*th male under sperm competition (${{Z}_{ik}}$) after his *k*th mate remated, was calculated as the proportion of the total potent sperm retained in the female after the two matings, which belonged to the *i*th male:


(11) \begin{eqnarray*}
{{Z}_{ik}} = \frac{{{{S}_{ik}}}}{{{{S}_{ik}} + {{A}_{ik}}\ }}.
\end{eqnarray*}

The total number of sperm from both males (*X*) in the female was thus written as


(12) \begin{eqnarray*}
{{X}_{ik}} = {{S}_{ik}} + {{A}_{ik}}.
\end{eqnarray*}

We calculated the number of offspring sired by the *i*th male ${{O}_{ik}}$ with the *k*th female as


(13) \begin{eqnarray*}
{{O}_{ik}} = {\mathrm{min\ }}({{X}_{ik}},{{E}_{ik}})*{{Z}_{ik}},
\end{eqnarray*}

where ${{E}_{ik}}$ is the total number of eggs the female allocates to be fertilized by the two males. To satisfy Fisher’s condition in the sperm competition systems, females allocated twice the number of eggs for fertilization compared with systems without sperm competition.

### Calculating the BG

BGs (${{\beta }_{\mathrm{ss}}}$) were calculated as the slope (regression coefficient) of the OLS linear regression, where male relative reproductive success ($r{{s}_i}$) was regressed on their relative mating success ($m{{s}_i}$):


(15) \begin{eqnarray*}
m{{s}_i} = \frac{{{\mathrm{M}}{{{\mathrm{S}}}_i}}}{{{{\mu }_{{\mathrm{MS}}}}\ }},
\end{eqnarray*}

(15) \begin{eqnarray*}
r{{s}_i} = \frac{{{\mathrm{R}}{{{\mathrm{S}}}_i}}}{{{{\mu }_{{\mathrm{RS}}}}\ }}, \mathrm{and}
\end{eqnarray*}

(16) \begin{eqnarray*}
{{\beta }_{\mathrm{ss}}} = {{r}_{{\mathrm{ms,\,rs}}}}\left( {\frac{{{{\sigma }_{\mathrm{rs}}}}}{{{{\sigma }_{\mathrm{ms}}}}}} \right),
\end{eqnarray*}

where ${{r}_{{\mathrm{ms}},\ {\mathrm{rs}}}}$ is the correlation between *ms* and *rs*, while ${{\sigma }_{\mathrm{rs}}}$ and ${{\sigma }_{\mathrm{ms}}}$ are their sample standard deviations. In each of our nine simulated scenarios within each of the four biological systems, we simulated 20 replicates with 2,000 males per replicate. ${{\mu }_{{\mathrm{MS}}}},$  ${{\mu }_{{\mathrm{RS}}}}$, and ${{\beta }_{\mathrm{ss}}}$ were calculated separately for each replicate within each scenario and system, and then averaged across the 20 replicates to obtain the final (mean) ${{\beta }_{\mathrm{ss}}}$ for each scenario within each system.

### Simulation assumptions

We made certain assumptions for the sake of simplicity:

- All traits for the focal male and females were sampled from a normal distribution.
- Focal males mated sequentially with females and without replenishment of ejaculates.
- Males in the population did not directly interact with each other.
- Males or females did not die during the simulation.
- There was no long term-sperm storage, and the *k*th females fertilized her allocated eggs immediately after the *i*th male (in the systems without sperm competition) or his sperm competitor (in systems with sperm competition) had copulated.
- The outcome of sperm competition was determined solely based on the relative proportion of a male’s potent sperm retained in the female. We did not model sperm offence–defense dynamics or precedence.
- Neither males nor females remated with the same partner.
- In the system with sperm competition, there was no variance in female degree of polyandry, and all females mated with two males.
- We did not covary male age or time with female rank, such that later females encounter males who are of an older age.

### Parametrization

For the low anisogamy system, we parametrized our traits based on data from *D. melanogaster* (Table 1), because it represents one of the lowest ratios of sperm:egg numbers in animals (Bjork & Pitnick, 2006). In *D. melanogaster*, the observed degree of anisogamy (sperm number reserves in male:egg number reserves in females) is ∼30:1, while it is 6:1 in *D. bifurca* and *D. hydei* (Bjork & Pitnick, 2006; Bjork et al., 2007). We chose 7.5:1 as the degree of low anisogamy for the sake of argument. For the high anisogamy system, we parametrized sperm and egg numbers (thus degree of anisogamy of 100,000 sperm:1 egg) based on data from red junglefowl (*G .gallus*). The standard deviation ($\sigma $) for all trait distributions was set as 20% of the mean.

### Data analysis

We simulated nine different scenarios in four biological systems, each across 20 replicates with 2,000 males per replicate. We first compared the BG obtained from the null scenario against each covariance scenario within a biological system. Under the assumption of additivity, the difference between the BG in the covariance scenario and the null scenario isolates the portion of the BG attributable solely to the confounding effect of the specified covariate. To do this, we first calculated the *rs* and *ms* for each male, and then ${{\beta }_{\mathrm{ss}}}$, separately for each of the 20 replicates within each scenario and system. Next, we created a linear model with Gaussian error distribution with ${{\beta }_{\mathrm{ss}}}$ as the dependent variable and scenario ID as the fixed effect (20 values per scenario), separately for each of the four systems. The null scenario was set as the reference to allow comparisons of the BG of other covariance scenarios against that of the null scenario. Using ANOVA (analysis of variance) test on the linear model, we additionally tested for overall differences in the means of the different scenarios within each system. We ensured that all our linear models met assumptions of normal distribution of residuals and homoscedasticity. Within each biological system, the distribution of offspring produced with a female through the mating sequence, and of MS, was similar across all scenarios (Supplementary Figures S2–S6).

Next, we used linear mixed models in the *lme4* package (Bates et al., 2014) to calculate partial BGs (i.e., BGs calculated as partial regression coefficients) that account for linear covariances between MS and other traits (following Henshaw et al., 2018, 2020), and tested whether these partial BGs were similar to the BG in the null model. These models were constructed to understand whether confounded relationships between MS and RS can still allow interpretation of the true strength of precopulatory sexual selection, if the confounding variable is accounted for. To do this, we only used data from the high anisogamy system with sperm competition because this is arguably the more common of the four systems observed. We fit models for each scenario separately. In a model with Gaussian error distribution, we first fit *rs* as our dependent variable, *ms* as the only fixed effect, and replicate ID as a random effect (to account for nonindependence between different males from the same replicate). We then compared the BG estimated in this model with one where all other traits (*Q, V, T, L, F*, and *P*) as well as *ms* were included as fixed effects. Since the null scenario had no covariances between traits, we predicted that adding these traits would not affect the BG compared with the simpler model with only *ms* as a fixed effect. Next, we created these linear models for each covariance scenario. First, we built a model with only *ms* as a fixed effect; second, a model with *ms* and the covarying trait as fixed effects; and third, a model with *ms* and all traits (*Q, V, T, L, F*, and *P*) as fixed effects. We predicted that the model with only MS as a fixed effect would produce an inflated or deflated BG compared with the BG of the null model, due to unaccounted covariances. In the second model, accounting for the covarying trait would give a partial BG similar to that measured in the null scenario. In the third model, adding noncovarying traits would not further alter the BG compared with the second model. Importantly, these partial regression coefficients provide an unbiased estimate of the total causal effect of MS on RS only when the included covariate is a confounder of the MS–RS relationship, not when it is a mediator (see Figure 1).

## Results

### Low anisogamy, no sperm competition

**Figure 2.**
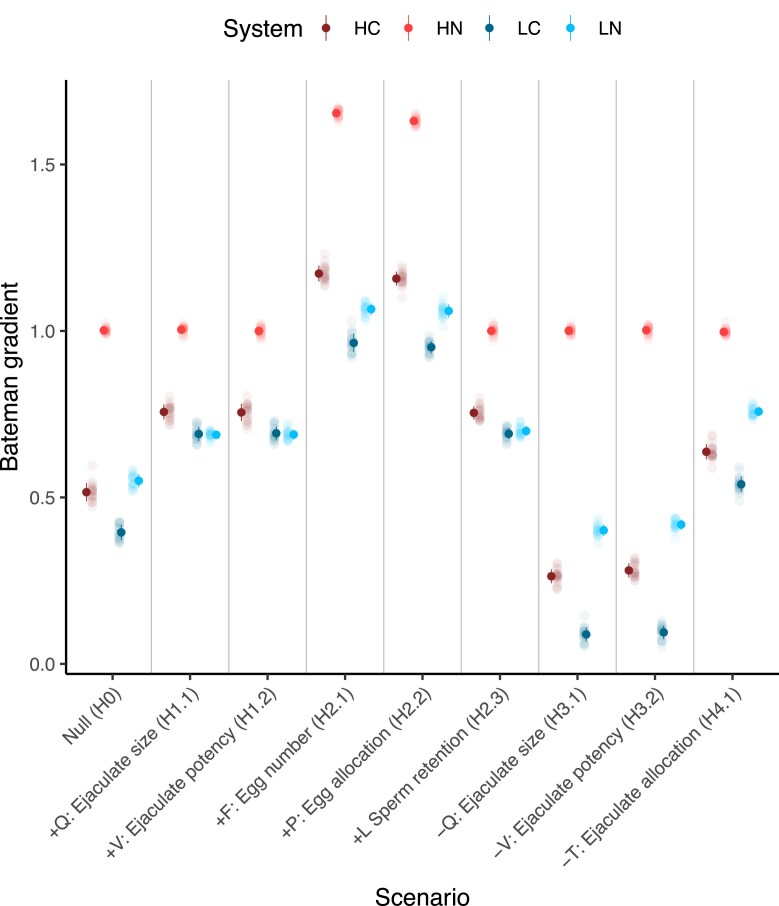
Bateman gradients (BGs) for each of the nine scenarios across the four biological systems. *Y*-axis represents the average increase in relative reproductive success when relative mating success increases by one unit. Dots show mean BG per scenario per system; error bars show standard deviations of the BG across 20 replicates (jittered). HC: high anisogamy with sperm competition; HN: high anisogamy without sperm competition; LC: low anisogamy with sperm competition; and LN: low anisogamy without sperm competition. *X*-axis ticks show the covarying trait name and symbol, the sign of covariance, and the scenario ID. See Supplementary Figures S7 and S8 for BGs represented using regressions.

Positive covariances between male MS and *Q* (starting ejaculate size), *V* (proportion of potent sperm), *F* (number of eggs in female), *P* (proportion of allocated eggs), or *L* (proportion of sperm retained) led to significantly steeper BG, while negative covariances between MS with *Q* or *V* resulted in significantly shallower BG (Figure 2, Supplementary Table S1, and Supplementary Figures S7 and S8), compared with the null scenario. Negative covariance between MS and *T* (proportion of stored sperm transferred when copulating) produced a significantly steeper BG than the null scenario.

**Figure 3.**
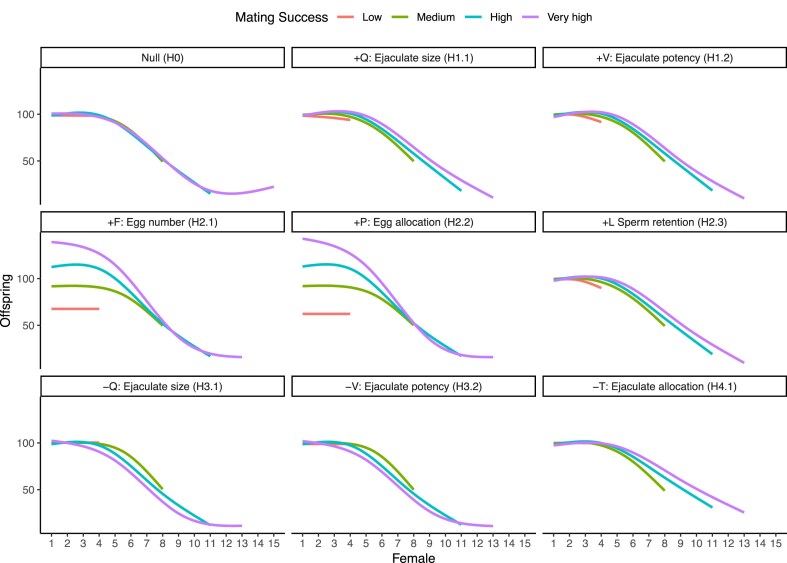
Effect of mating success (MS) on the number of offspring a focal male produces with each female (rank) in his mating sequence. Panel labels correspond to each scenario in the system with low anisogamy and no sperm competition. Data combined from all 20 replicates. MS binned into categories for ease of visualization. Lines show means of 20 replicates, constructed as gam smooths with four knots in ggplot. Facet titles show the covarying trait name and symbol, the sign of covariance, and the scenario ID.

Visual inspection (Figure 3) of offspring production through the mating sequence revealed that in the null scenario (H0), patterns of offspring production were the same for males with different MS. In scenarios where MS covaried positively with *Q, V*, or *L* (H1.1, H1.2, and H2.3), males with higher MS had shallower rates of decline in offspring production compared with males with lower MS. Conversely, in scenarios where MS covaried negatively with *Q* or *V* (H3.1 and H3.2), males with higher MS experienced steeper declines in offspring production through the mating sequence. When MS and *T* negatively covaried (H4.1), males with higher MS experienced shallower rates of declines in offspring production through the mating sequence compared with males with lower MS. When MS covaried positively with *F* or *P*, males with higher MS consistently produced more offspring than males with lower MS, irrespective of female rank (H2.1 and H2.2).

### Low anisogamy, with sperm competition

**Figure 4.**
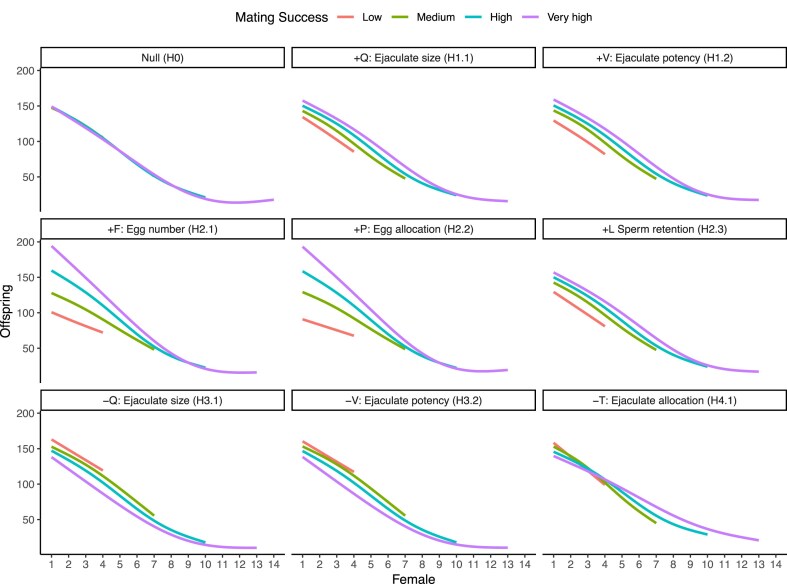
Effect of mating success (MS) on the number of offspring a focal male produces with each female (rank) in his mating sequence. Panel labels correspond to each scenario in the system with low anisogamy and sperm competition. MS binned into categories for ease of visualization. Lines show means of 20 replicates, constructed as gam smooths with four knots in ggplot. Facet titles show the covarying trait name and symbol, the sign of covariance, and the scenario ID.

Positive covariances between MS and *Q, V, L, F*, or *P* led to significantly steeper BG than the null scenario (Figure 2, Supplementary Table S2, and Supplementary Figures S7 and S8), while negative covariances of *MS* with *Q* or *V* produced a shallow BG. Negative covariance between MS and *T* resulted in a significantly steeper BG than the null scenario (Supplementary Table S2). Patterns of offspring production across the mating sequence were the same for focal males with different MS in the null scenario (Figure 4). However, when male MS covaried positively with *Q, V, L, F*, or *P*, males with higher MS consistently produced more offspring with each female compared with males with lower MS, irrespective of female rank in the mating sequence. On the other hand, when MS covaried negatively with *Q* or *V*, males with higher MS consistently produced fewer offspring with each female irrespective of female rank, compared with males with lower MS. Additionally, when MS covaried positively with *T*, males with higher MS produced fewer offspring with females early in the sequence, but more offspring with females later in the sequence, compared with males with lower MS.

### High anisogamy, no sperm competition

**Figure 5.**
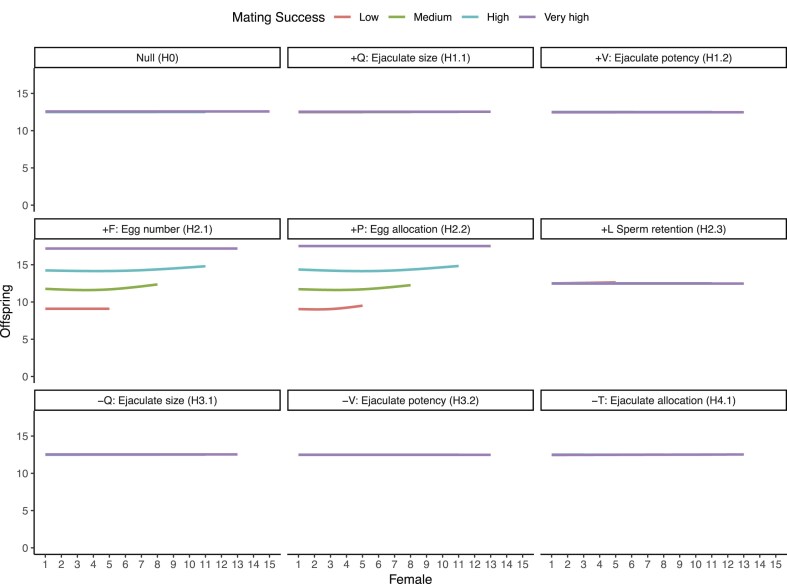
Effect of mating success (MS) on the number of offspring a focal male produces with each female (rank) in his mating sequence. Panel labels correspond to each scenario in the system with high anisogamy and no sperm competition. MS binned into categories for ease of visualization. Lines show means of 20 replicates, constructed as gam smooths with four knots in ggplot. Facet titles show the covarying trait name and symbol, the sign of covariance, and the scenario ID. The increase in offspring production with later females is a consequence of each binned category plotted as an average of multiple MS values.

Positive covariances only between MS and *F* or *P*, led to significantly steeper BG than the null scenario (Figure 2, Supplementary Table S3, and Supplementary Figures S7 and S8). However, BG from the null scenario did not significantly differ from BGs of scenarios in which MS covaried with *Q, V, T*, or *L* (Supplementary Table S3). Patterns of offspring production through the mating sequence were modulated by MS only when MS covaried positively with *F* or *P* (Figure 5). Here, males with higher MS consistently produced more offspring than males with lower MS, irrespective of female rank. In contrast to the systems with low anisogamy, offspring production of focal males did not decline through the mating sequence in the high anisogamy system without sperm competition.

### High anisogamy with sperm competition

Positive covariances between MS and ejaculate size (*Q*, H1.1), ejaculate potency (*V*, H1.2), sperm retained by females (*L*, H2.3), female egg numbers (*F*, H2.1), or egg allocation (*P*, H2.2), led to significantly steeper BG, while negative covariance between male MS and *Q* (H3.1) or *V* (H3.2) led to a significantly shallower BG, than the null scenario (Figure 2, Supplementary Table S4, and Supplementary Figures S7 and S8). Negative covariance between MS and the proportion of a male’s stored sperm transferred to a female (*T*, H4.1), led to significantly steeper BG than the null scenario. Offspring production through the mating sequence was not modulated by MS in the null scenario. However, in all covariance scenarios, MS modulated offspring production (Supplementary Figure S9), and did so in a similar way as in the low anisogamy system with sperm competition.

**Figure 6.**
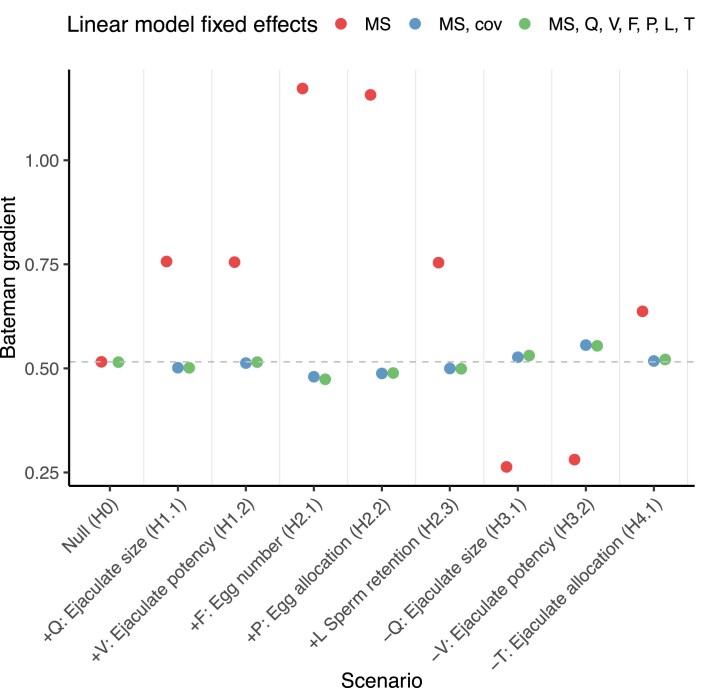
Comparison of Bateman gradients (BGs) vs. partial BG that account for confounding variables, across nine different scenarios in the system with high anisogamy and sperm competition. *X*-axis ticks show the covarying trait name and symbol, the sign of covariance, and the scenario ID. In red, BG from linear models where only relative mating success (*ms*) is included as a fixed effect, with relative reproductive success as the dependent variable. In blue, partial BG from models where only the confounding variable (cov) for that scenario along with *ms* are included as fixed effects. In green, partial BG from linear models where all traits (including the cov) and *ms* are included as fixed effects. Mean estimates of the BG from the linear models shown as dots (*N* = 2000 males per scenario across 20 replicates). Dotted horizontal line shows the BG for the null scenario.

We also evaluated whether adding the confounding trait in each scenario as a fixed effect led to the estimated BG being closer to the null scenario, thus better representing the causal influence of MS on RS (Figure 6). In the null scenario where no covariances existed, including additional traits did not alter the influence of MS on RS. However, in scenarios where MS covaried with another trait, the partial BG—obtained by including the covarying trait as a linear fixed effect—led to more similar estimates to the BG from the null scenario compared with models that did not include the covarying trait (Figure 6).

## Discussion

We conducted simulations to quantify the influence of specific covariances between MS and other male and female reproductive traits on RS, to understand which common sources of confounding might be especially problematic when interpreting the strength of sexual selection using the BG. We found that, generally, covariances between male MS and other variables modulated the BG, but this was dependent on the identity of the confounding variable (aim 1) and the biological system (aim 2). We also demonstrated that recording the mating sequence and associated reproductive output provides a visual diagnostic tool for assessing the presence and type of confounding (aim 3), complementing the quantitative approach using partial BGs (aim 4).

The BG is typically used to interpret the strength of precopulatory sexual selection, i.e., the potential improvement in fitness obtained by acquiring more mates (Wade & Shuster, 2005). Males, due to having steeper BGs, are typically hypothesized to experience more intense precopulatory sexual selection than females, leading to predictions of males competing for mates and being promiscuous, more than females. However, consistent with previous critiques (Anthes et al., 2010, 2017; Henshaw et al., 2018; Tang-Martinez, 2016), we show that without accounting for confounded effects of MS on RS when present, using the BG to compare the strength of sexual selection for mate acquisition can be misleading (also see Anthes et al., 2010, 2017; Kokko & Jennions, 2023; Pischedda & Rice, 2012 who discuss this). Confounded BGs reflect a composite of precopulatory selection, postcopulatory selection, and/or selection on mate quality. Modulation of the BG due to such covariances has important implications for quantifying sex differences in sexual selection. For instance, if a steeper BG in males than females (as typically predicted) in a species is caused by covariances between male MS and ejaculate traits rather than a causal effect of MS on RS, then the difference in BGs between sexes may not reliably indicate stronger precopulatory sexual selection on males than females unless partial BGs are used.

A fundamental puzzle in sexual selection is why male BGs vary within (e.g., *D. melanogaster*, reviewed in Davies et al., 2023) as well as between species (Anthes et al., 2017). Our simulations provide some explanations for interspecific and intraspecific variation in BG, solely based on the presence or absence of certain covariances in specific systems. For instance, in populations where males vary in their condition, high condition males might have more competitive ejaculates as well as higher MS, compared with low condition males (Puniamoorthy et al., 2012; Reznick et al., 2000). Such covariances due to condition dependence (e.g., Hare & Simmons, 2022; Janicke et al., 2015; Mehlis et al., 2015; Morimoto et al., 2016) would lead to the measured BG being steeper in these populations compared with populations of the same species where male condition is more homogeneous. On the other hand, in species with alternative mating tactics, negative covariances between male MS and ejaculate size, quality, or allocation (e.g., Alonzo & Warner, 2000; Durant et al., 2016; Froman et al., 2002; Gage et al., 1995; Klaus et al., 2011; Lupold et al., 2014; Preston et al., 2001; Saunders & Shuster, 2020; Warner et al., 1995) could lead to a shallower male BG compared with species without such tactics. For instance, males in dominant roles in species with alternative mating tactics often achieve similar overall RS as males in subordinate roles (Da Silva et al., 2024). In such scenarios, the relationship between MS and RS could be curvilinear (see Supplementary Figure S8) with male fitness being maximized at average MS. The BG might also be inflated in species with condition-based assortative mating, where males in high condition (who have high MS) mate with females in high condition (who have high fecundity) (e.g., Collet et al., 2014; Gerlach et al., 2012; Greenway et al., 2021; Kraak & Bakker, 1998). Importantly, when populations or species differ in their BGs due to the presence of certain variables confounding the effect of MS on RS, the BGs cannot be interpreted as different strengths of precopulatory sexual selection on acquiring mates between these populations/species.

Variation in BGs between different species could also be due to differences in anisogamy or sperm competition between taxa. Anisogamy underpins the evolution of divergent reproductive strategies, and an increase in the degree of anisogamy is predicted to lead to a steeper BG for the sex with more numerous gametes (Henshaw et al., 2022; Janicke, 2024; Jones et al., 2002; Lehtonen, 2022; Lehtonen & Parker, 2024). In our models, the impact of confounding variables on the BG not only depended on the identity of the variable, but also on the system. In the absence of sperm competition, males in high anisogamy systems gained more fitness benefits by improving their MS, compared with males in low anisogamy systems (Figure 2). However, if sperm competition is present within high anisogamy systems, the differences in BG between high and low anisogamy systems becomes less apparent (Figure 2). Importantly, confounding effects of male ejaculate traits did not impact the BG in high anisogamy systems without sperm competition. These findings generally agree with Arnold (1994), who suggested that a positive, linear relationship between MS and RS for males can only be expected when their RS is not limited by gamete production rate (e.g., in high anisogamy systems without sperm competition).

The importance of partitioning the measured BG into episodes of selection on precopulatory vs. postcopulatory traits has been previously emphasized (Anthes et al., 2017; Evans & Garcia-Gonzalez, 2016; Pelissie et al., 2014). Our results show that it is difficult to disentangle precopulatory from postcopulatory episodes of sexual selection when using the BG, a correlational (rather than process-based) metric. By including postcopulatory processes such as cryptic female choice via differential sperm retention or female fecundity stimulation (see Eberhard, 2015; Firman et al., 2017), and their covariances with MS, our simulations highlight the under-appreciated role of female agency in impacting male BG. Our simulations also demonstrate that not all mechanisms of cryptic female choice are equal in modulating the BG. Generally, we found that the mechanism of fecundity stimulation (H2.2) had a greater impact on the BG than sperm ejection (H2.3), underscoring the importance of the identity of the confounding variable.

While our simulations demonstrate that covariances between MS and other ejaculate or female traits can severely distort interpretation of the strength of sexual selection, this does not diminish utility of the BG. We suggest two diagnostic methods for researchers working with empirical data, to assess, and in certain cases, account for confounded BG. First, we show that when female mating rank is recorded, visually examining offspring production of males with each female can allow identification of the presence, and importantly, the type of underlying covariances (see Figures 3–6). In the absence of covariances, no systematic differences should exist in the rate of change or the intercept for offspring production across the mating sequence, between males with varying MS (see Sanghvi et al., 2025b who show this). However, when there are differences only in intercepts, we show that these could indicate a confounded effect of ejaculate quality or female fecundity. On the other hand, when there are differences in intercepts and slopes, this might indicate covariances between male MS and ejaculate allocation. For studies where the mating order of females is recorded, the presence of confounds can be statistically tested by fitting


\begin{eqnarray*}
{{O}_{ik}}\ \sim\ {\mathrm{ran}}{{{\mathrm{k}}}_{ik}} + {\mathrm{M}}{{{\mathrm{S}}}_i} + {\mathrm{ran}}{{{\mathrm{k}}}_{ik}}:{\mathrm{M}}{{{\mathrm{S}}}_i} + {{b}_i},
\end{eqnarray*}

where ${{O}_{ik}}$ is the number of offspring produced by each focal male mated with each female, rank is the rank of the female encountering the male in his mating sequence, and ${{b}_i}$ is the random effect of male ID to control for male-level nonindependence in offspring production. Here, a significant effect of ${\mathrm{ran}}{{{\mathrm{k}}}_{{\mathrm{ik}}}}:{\mathrm{M}}{{{\mathrm{S}}}_i}$ or of ${\mathrm{M}}{{{\mathrm{S}}}_i}$ might suggest presence of covariances between MS and other variables that indirectly influence male RS. Conceptually, this process resembles selective disappearance, where the death of individuals over time is dependent on their reproductive quality, leading to a biased subset of individuals at later time ages (Bouwhuis et al., 2009). Second, when researchers can collect data on variables that covary with RS and MS, studies could account for these confounds and interpret the strength of precopulatory sexual selection using the partial BG (following Henshaw et al., 2018). However, differentiating a confounding from a mediating variable might not always be possible (Arif & MacNeil, 2023), and only confounders should be adjusted for, whereas mediators should not, when estimating total effects (Supplementary Figure S10). One way to identify a confounding variable (a result of between-male variation) from a mediating variable (a result of within-male variation), is by inspecting reproductive output through mating sequence as explained above. While Anthes et al. (2017) caution against using partial BGs, we show that in the absence of better alternatives, and when the influence of the confounding variable is additive and linear, the partial BG represents the causal influence of MS on RS. While our simulations control causal structure, in real data it is difficult to account for all confounding variables. Applying these findings to empirical systems is thus challenging. However our study identifies some common variables that might be confounding, and we quantify their influence to highlight in which systems these are especially problematic as a guide for future research.

Fitness is not only determined by the number of offspring produced, but also by the reproductive value of each offspring (Grafen, 2020). Therefore, the strength of precopulatory sexual selection on acquiring mates will be determined by the influence of MS on RS, as well as of MS on offspring quality (Kokko & Jennions, 2008) and offspring lifetime RS. This distinction is crucial when MS influences offspring quality and RS in contrasting ways (Arnold, 1994; Queller, 1997). For example, when males who mate with more females produce more offspring but are less able to provision each offspring thereby reducing offspring quality (e.g., Lynch, 2016), males with average MS would likely produce the most numbers of grand offspring despite producing fewer offspring than males with the highest MS (reviewed in Stiver & Alonzo, 2009). Here, despite the BG being positive and the relationship between MS and RS being linear, interpreting the BG as evidence of strong sexual selection on mate acquisition would be misleading. This is because selection is likely to favor a strategy that maximizes the production of grand offspring produced rather than merely of offspring (i.e., RS). Similarly, in females, polyandry (i.e., increasing female MS) could confuse the paternity of males, leading to various males provisioning a female’s offspring, which consequently improves offspring quality (Hosken & Stockley, 2003). Here, despite polyandry not directly improving female RS and the BG being shallow, polyandry might still lead to increased numbers of grand offspring via improved offspring quality (reviewed in Jennions & Petrie, 2000; Parker & Tang-Martinez, 2005; Simmons, 2005). In such a case, interpreting a shallow female BG as weak precopulatory sexual selection would be misleading. We therefore recommend that covariances between MS and offspring quality, in addition to those between MS and RS (i.e., offspring number), be considered within the BG framework.

Our simulations were limited in their complexity because we did not manipulate degree of polyandry, sperm precedence, male and female lifespans, sperm storage duration, the rate of ejaculate and egg replenishment, offspring survival, or offspring RS. Future studies could test how covariances between MS and these traits might influence the BG and the strength of precopulatory sexual selection. For instance, if males who are more attractive (and thus have higher MS) also possess genes that improve offspring survival until detection (e.g., Head et al., 2005), BG would be higher than expected. Our simulations focused only on male BG. However, female BG can also be influenced by covariances between RS and other male or female traits (e.g., Collett et al., 2014; Fromonteil et al., 2023; Gerlach et al., 2012). Future studies could simulate various male- and female-specific covariances in polygynandrous systems to test whether sex differences in the strength of sexual selection become less apparent after accounting for such covariances (by using partial BGs). Finally, we emphasize that studies which experimentally manipulate MS are lacking and encourage researchers to compare BGs obtained from experimental manipulation of MS (e.g., Andrade & Kasumovic, 2005) vs. from spontaneously available variation in MS, to understand how covariances might modulate the BG in nature.

## Conclusions

Confounded BG estimates are a known issue in the quantification of precopulatory sexual selection. However, our study is the first to quantify the scale of this problem by identifying multiple, biologically common, confounding variables and covarying these with MS artificially to demonstrate how these can distort interpretation of sexual selection when using the BG (aim 1). Importantly, our simulations can distinguish more problematic covariances (e.g., with female fecundity) from less problematic ones (e.g., ejaculate phenotype), and identify which systems might be less vulnerable to the known confounded effects of certain variables (aim 2). Our study adds to the growing body of research advocating for a more nuanced understanding and application of the BG (Anthes et al., 2017; Henshaw et al., 2016; Kokko & Jennions, 2023; Parker & Tang-Martinez, 2005). We illustrate that BG measurements that fail to account for covariances that confound (rather than mediate) the effect of MS on RS, can oversimplify its interpretation and misattribute the magnitude of selection solely to precopulatory competition for mates. Importantly, we provide diagnostic guidelines and hypothesis generating tools for studies to qualitatively and quantitatively assess the presence, magnitude, and type of covariances when mating orders are recorded (aim 3), and account for measured confounding variables (aim 4) to better interpret the BG. Our simulations on how different mechanisms cause variation in the BG, improve understanding of what the BG represents and what it does not and provide novel insights into sexual selection.

## Supplementary Material

qpaf127_Supplemental_File

## Data Availability

Simulation code and generated simulation data can be found at Open Science Framework DOI:10.17605/OSF.IO/WPE8M. Data and code are also available on DRYAD: https://doi.org/10.5061/dryad.mcvdnckbc. Open AI ChatGPT 4.0 was used to streamline the code.
